# Supplementary material for: Shifting Compasses: A Qualitative Study of Lived Experiences Driving Perioperative Nurses to Leave the Profession Post COVID-19
Source: Healthcare (Basel). 2025 Feb 11;13(4):391. doi: 10.3390/healthcare13040391 (PMC11855720; doi:10.3390/healthcare13040391)
Supplement: Supplementary file 1 [file healthcare-13-00391-s001.zip › healthcare-3463146-supplementary.pdf]

Supplementary File S1: COREQ checklist

Consolidated criteria for reporting qualitative studies (COREQ): 32-item checklist

Developed from:

Tong A, Sainsbury P, Craig J. Consolidated criteria for reporting qualitative research (COREQ): a 32-item checklist for interviews and focus groups. International Journal for Quality in Health Care. 2007. Volume 19, Number 6: pp. 349 – 357

Item No	Guide Questions/Description	Reported on Page #
Domain 1: Research team and reflexivity		
Personal Characteristics		
1. Interviewer/ facilitator	Which author/s conducted the interview or focus group?	Pg 3
2. Credentials	What were the researcher's credentials? E.g., PhD, MD	Pg 4
3. Occupation	What was their occupation at the time of the study?	Pg 4
4. Gender	Was the researcher male or female?	N/A
5. Experience and training	What experience or training did the researcher have?	Pg 4
Relationship with participants		
6. Relationship established	Was a relationship established prior to study commencement?	Pg 4
7. Participant knowledge of the interviewer	What did the participants know about the researcher? e.g. personal goals, reasons for doing the research?	Pg 4
8. Interviewer characteristics	What characteristics were reported about the interviewer/facilitator? e.g. Bias, assumptions, reasons and interests in the research topic	Pg 4
Domain 2: study design		
Theoretical framework		
9. Methodological orientation and Theory	What methodological orientation was stated to underpin the study? e.g. grounded theory, discourse analysis, ethnography, phenomenology, content analysis	Pg 3

Item No	Guide Questions/Description	Reported on Page #
Participant selection		
10. Sampling	How were participants selected? e.g., purposive, convenience, consecutive, snowball	Pg 3
11. Method of approach	How were participants approached? e.g., face-to-face, telephone, mail, email	Pg 3-4
12. Sample size	How many participants were in the study?	Pg 3
13. Non-participation Setting	How many people refused to participate or dropped out? Reasons?	Pg 3
14. Setting of data collection	Where was the data collected? e.g., home, clinic, workplace	Pg 3
15. Presence of nonparticipants	Was anyone else present besides the participants and researchers?	Pg 4
16. Description of sample	What are the important characteristics of the sample? e.g. demographic data, date	Pg 4 and Pg 5-6 (Table 2)
Data collection		
17. Interview guide	Were questions, prompts, and guides provided by the authors? Was it pilot tested?	Pg 3-4
18. Repeat interviews	Were repeat interviews carried out? If yes, how many?	N/A
19. Audio/visual recording	Did the research use audio or visual recording to collect the data?	Pg 3-4
20. Field notes	Were field notes made during and/or after the interview or focus group?	N/A
21. Duration	What was the duration of the interviews or focus group?	Pg 3-4
22. Data saturation	Was data saturation discussed?	Pg 4
23. Transcripts returned	Were transcripts returned to participants for comment and/or correction?	Pg 3-4
Domain 3: analysis and findings		
Data analysis		
24. Number of data coders	How many data coders coded the data?	Pg 4-5
25. Description of the coding tree	Did the authors provide a description of the coding tree?	N/A
26. Derivation of themes	Were themes identified in advance or derived from the data?	Pg 8

Item No	Guide Questions/Description	Reported on Page #
27. Software	What software, if applicable, was used to manage the data?	Pg 4-5
28. Participant checking	Did participants provide feedback on the findings?	N/A
**Reporting**		
29. Quotations presented	Were participant quotations presented to illustrate the themes/findings? Was each quotation identified? e.g., participant number	Pg 8-11
30. Data and findings consistent	Was there consistency between the data presented and the findings?	Pg 12-14
31. Clarity of major themes	Were major themes clearly presented in the findings?	Pg 13-14
32. Clarity of minor themes	Is there a description of diverse cases or a discussion of minor themes?	Pg 11-14
